# Reviewed article: Nerbass FB, Hanauer MA, Silva IF, Costa TF, Silva RM, Fabre L, Vieira MA, Calice-Silva V. Profile, follow-up adherence and progression of people with advanced chronic kidney disease in specialized care centers: a retrospective study, Santa Catarina state, Brazil, 2021-2024. Epidemiol Serv Saude. 2026:35;e20250690.

**DOI:** 10.1590/S2237-96222026v35e20250690.b

**Published:** 2026-04-10

**Authors:** Nathalia Sernizon Guimarães

**Affiliations:** 1 Universidade Federal de Minas Gerais, Belo Horizonte, MG, Brazil

## First round comments

Agradecemos a submissão do manuscrito e reconhecemos a relevância do tema abordado, especialmente considerando os desafios enfrentados pelo SUS no manejo da doença renal crônica (DRC). No entanto, após avaliação por pareceristas ad hoc, identificamos limitações metodológicas, estruturais e analíticas que comprometem a robustez científica do trabalho em sua versão atual. Assim, solicitamos revisões substanciais com base nas seguintes observações consolidadas:

Quanto a estruturação geral: O texto apresenta erros gramaticais e tipográficos recorrentes (ex.: “doença renal crônia”, “especiailzado”), e diversas passagens são vagas, pouco explicativas ou genéricas, comprometendo a clareza e a fluidez do texto. Uma revisão minuciosa da redação é fundamental.

O manuscrito carece de uma justificativa clara para a pergunta de pesquisa e de uma definição mais precisa sobre qual lacuna no conhecimento pretende preencher. A introdução, embora contextualize a DRC como problema de saúde pública, não conecta adequadamente esse panorama com os objetivos do estudo.

Recomenda-se a reestruturação de trechos que utilizam linguagem imprecisa, como “população urbana selecionada” e “grande banco de dados”, substituindo por descrições mais específicas e científicas.

O uso da sigla DRC deve ser padronizado ao longo de todo o texto.

Quanto a introdução: Sugere-se maior fluidez entre os parágrafos e inclusão dos critérios clínicos que fundamentam a definição dos estágios 4 e 5 da DRC, já que esses compõem o recorte da amostra. Ainda, deve-se evitar termos desatualizados como “portador” ao se referir a condições de saúde.

Quanto aos métodos os revisores consideraram que, a seção de métodos é uma das mais frágeis do manuscrito. Faltam detalhes sobre as unidades incluídas (quais são, onde estão localizadas, quantas existem no estado, e por que foram escolhidas).

Os critérios de inclusão e exclusão devem ser claramente explicitados, assim como a caracterização da população estudada e o período de coleta dos dados.

É necessário indicar se as medidas de filtração glomerular foram obtidas em dois momentos distintos com intervalo ≥ 3 meses, como recomendado para diagnóstico de DRC.

A padronização entre os centros de atendimento, em termos de protocolo de cuidado e coleta de dados, não está clara.

A ausência de descrição ética na seção de métodos (apesar de constar após o resumo) deve ser corrigida.

A data de aprovação no comitê de ética (junho de 2025) levanta dúvidas sobre a viabilidade temporal da coleta, análise e submissão em tão curto prazo.

É necessário justificar os critérios para uso de média/DP ou mediana/IIQ e apresentar quais testes de normalidade foram empregados. O uso de testes estatísticos, como Wilcoxon, precisa de fundamentação mais clara. Além disso, sugere-se a inclusão de testes de diferença de proporções para variáveis categóricas.

A principal limitação metodológica apontada foi o acompanhamento de menos de 50% dos participantes, sem análise dos fatores associados à evasão. A ausência dessa análise compromete o entendimento do perfil dos pacientes que abandonaram o seguimento - grupo potencialmente mais vulnerável à progressão da DRC.

A ausência de modelos estatísticos para controle de fatores de confusão também fragiliza as conclusões. Variáveis sociodemográficas e clínicas foram coletadas, mas não foram incorporadas a análises ajustadas.

A inferência de causalidade entre seguimento especializado e preservação da função renal não é sustentada pelo delineamento retrospectivo. Recomendamos reformular essas afirmações de forma mais cautelosa e ancorada na literatura.

Quanto aos resultados, Há inconsistências nos dados apresentados, como os valores da TFG na Tabela 2 (“22 (18-7)”), que parecem conter erros de digitação. Isso também se aplica à Figura 1, que precisa de revisão no título e apresenta informação redundante.

O número absoluto e a proporção de pacientes segundo o estadiamento da doença devem ser apresentados com clareza.

A categorização das variáveis precisa ser mais criteriosa: por exemplo, considerar os pontos de corte da OMS para adultos e da OPAS para idosos no IMC, e explicitar as categorias incluídas como “outras” em raça/cor da pele.

A variável “estado nutricional” tem excesso de subcategorias pouco exploradas na discussão, o que enfraquece a análise.

A discussão é excessivamente descritiva e pouco propositiva. Recomenda-se aprofundar a análise dos achados, especialmente sobre barreiras organizacionais ao acompanhamento (ex.: agendamento, transporte, tempo de espera) e impactos na adesão.

Sugere-se incluir implicações para o SUS e destacar a importância da atenção especializada no manejo da DRC.

Informações fisiopatológicas já consolidadas na literatura não agregam novidade ao manuscrito. Recomendamos que os autores avancem na interpretação crítica e relevância dos dados no contexto local.

Por fim, a conclusão deve refletir melhor os limites do delineamento e evitar extrapolações não sustentadas pelos dados apresentados. 

Recommendation: Major revision

## Second round comments

Agradecemos pela vasta revisão e melhoria do manuscrito. Gostaríamos de solicitar, de forma cordial, que revisem a escrita científica do texto, garantindo maior clareza, precisão e concisão, conforme as normas acadêmicas habituais.

Além disso, no trecho referente ao objetivo do estudo, peço que considerem substituir o termo “avaliação” por “descrição” ao tratar da adesão, de modo a refletir de forma mais adequada a natureza do trabalho apresentado.

Recommendation: Minor revision

